# From Samples to Insights into Metabolism: Uncovering Biologically Relevant Information in LC-HRMS Metabolomics Data

**DOI:** 10.3390/metabo9120308

**Published:** 2019-12-17

**Authors:** Julijana Ivanisevic, Elizabeth J. Want

**Affiliations:** 1 Metabolomics Platform, Faculty of Biology and Medicine, University of Lausanne, Rue du Bugnon 19, 1005 Lausanne, Switzerland; 2 Section of Biomolecular Medicine, Department of Metabolism, Digestion and Reproduction, Faculty of Medicine, Imperial College London, London SW7 2AZ, UK

**Keywords:** untargeted metabolomics, liquid chromatography–mass spectrometry (LC-MS), metabolism, experimental design, sample preparation, data processing, metabolite identification, univariate and multivariate statistics, metabolic pathway and network analysis

## Abstract

Untargeted metabolomics (including lipidomics) is a holistic approach to biomarker discovery and mechanistic insights into disease onset and progression, and response to intervention. Each step of the analytical and statistical pipeline is crucial for the generation of high-quality, robust data. Metabolite identification remains the bottleneck in these studies; therefore, confidence in the data produced is paramount in order to maximize the biological output. Here, we outline the key steps of the metabolomics workflow and provide details on important parameters and considerations. Studies should be designed carefully to ensure appropriate statistical power and adequate controls. Subsequent sample handling and preparation should avoid the introduction of bias, which can significantly affect downstream data interpretation. It is not possible to cover the entire metabolome with a single platform; therefore, the analytical platform should reflect the biological sample under investigation and the question(s) under consideration. The large, complex datasets produced need to be pre-processed in order to extract meaningful information. Finally, the most time-consuming steps are metabolite identification, as well as metabolic pathway and network analysis. Here we discuss some widely used tools and the pitfalls of each step of the workflow, with the ultimate aim of guiding the reader towards the most efficient pipeline for their metabolomics studies.

## 1. Introduction

It is assumed that metabolite identification remains a major challenge in untargeted mass spectrometry (MS)-based metabolomics. Is this indeed true? Should there be greater effort to design experiments in a smarter, more streamlined way, and to know how to reduce noise and redundancy in untargeted metabolomics datasets? For example, a meta-analysis comparative strategy can be used, where several pairwise comparisons are performed (with the same control group), followed by second-order or meta-analysis to prioritize the identification of the shared deregulated metabolites [1,2]. Here, we provide tips on how to design metabolomics experiments in an optimal way, considering sample size, confounders, and bias. We discuss important factors in sample preparation and describe how preparation approaches should be tailored to each biofluid or tissue. Methods should be simple, reproducible, and inexpensive, while preparation steps should not be biased for or against specific analytes, in order to maximize metabolome and/or lipidome coverage. We also summarize different liquid chromatography–mass spectrometry (LC-MS) strategies in order to acquire high quality MS and MS/MS data (reversed phase (RP) LC and hydrophilic interaction liquid chromatography (HILIC) coupled to full scan high resolution (HR) MS data-dependent and data-independent acquisition (DDA and DIA)), while maximizing the metabolome and lipidome coverage, parameters to pay attention to for data pre-processing, and, specifically, feature annotation. Also covered are which criteria to use for data filtering (quality control, chemical and informatic noise removal, etc.), how to apply statistical analysis in the best way, how to facilitate metabolite identification (using computational approaches) and how to translate the results in a biochemically relevant context (metabolite set enrichment analysis (MSEA), overrepresentation analysis (ORA), metabolic network analysis). We emphasize the importance in metabolomics studies of employing quality control (QC) strategies. QC samples, typically a pool of all study samples, can be used to both condition the analytical column and to monitor stability throughout the run. Expanding the polar metabolome and lipidome coverage, removal of noise and redundancy, and consideration of metabolic capacities of a model organism (i.e., biochemical reactions that can be performed by the specific organism, species, genus, etc.) are essential for generation of well-founded hypotheses from untargeted assays. We show how the mass analyzer for untargeted assays should harness high mass accuracy and resolution, and the ability to perform fragmentation or MS/MS experiments for structural elucidation. Many different software exist for the extraction of peaks (metabolite features) from the data, the deconvolution of such data, and the subsequent analysis in both multivariate and univariate ways. There are many statistical tools available, which aim to streamline and aid interpretation, of which we endeavor to summarize and evaluate some of the most commonly used. Finally, we highlight the lack of quantitative data and the need to validate these data-driven hypotheses using targeted quantification, with a focus on identified biochemical pathways associated with phenotype. These analyses will allow to go towards more mechanistic insights and, most importantly, allow for cross-laboratory and -study comparisons for intelligent data re-usage.

## 2. Results

### 2.1. Considerations for Experimental Design

**Figure 1:**
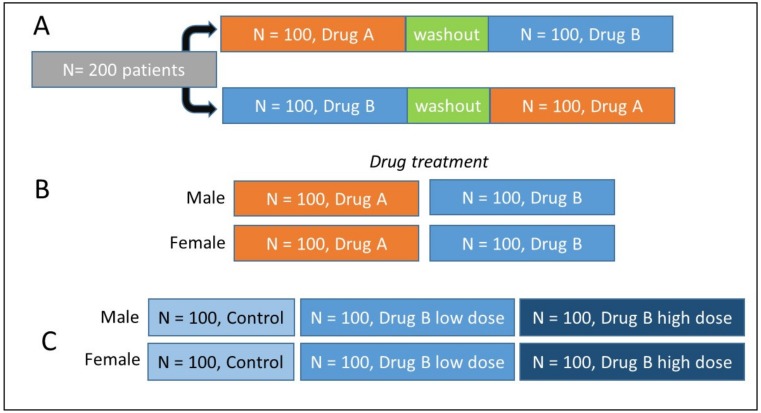
Common experimental designs. (**A**) Cross-over design involving a large patient cohort. Two drugs are administered sequentially to each patient, with a crucial washout period between each drug to enable the effects of each drug to be elucidated. (**B**) Factorial design, where both the gender of the subject and effect of the drug are being studied. (**C**) Common cross-sectional design in metabolomics studies, comparing controls and two drug dose levels in both genders.

Before starting any metabolomics study, it is important to consider the question(s) being asked. Many metabolomics studies are complex in design and may incorporate several classes, e.g., control subjects versus those receiving low and high dose of a drug (Figure 1), healthy subjects versus those with a benign condition and cancer (maybe several stages). It is vital that the study is designed to maximize useful information, whilst keeping costs and animal usage to a minimum. Ideally, you are aiming for the smallest number of experiments needed to produce the maximum amount of data and achieve precision, whilst addressing power and effect size, and accounting for confounding factors [3]. However, it is challenging to calculate the appropriate sample size for untargeted metabolomics studies, as metabolite changes are typically unknown and may be numerous. Further, the high dimensionality of the data and the large degree of correlation between the variables (metabolite features) adds to the complexity of the issue. Ideally, a pilot study should be conducted in order to gain an understanding of the expected effects; however, these are rarely performed due to logistical reasons (sample availability, cost, animal usage, ethics, etc.). Software such as MetaboAnalyst can aid in these calculations if pilot data are available [4]. Recently, Ebbels, et al. [5] proposed an approach to circumvent the need for obtaining preliminary data by using a multivariate simulation approach. Also publicly available is MetSizeR, which uses information from both the metabolomics experiment and the data analysis technique to simulate pilot data from a statistical model (where two groups are present). In order to estimate the required sample size, permutation-based techniques are applied to these simulated data [6]. Also important to consider is the nature of the experiment and the type of samples being analyzed. For instance, when using cell models, conditions can be tightly controlled, and thus sample numbers kept to a minimum (e.g., five replicates). Animal studies are also subject to fairly tight control in terms of age, housing, diet, underlying disease, etc. Therefore, for ethical and practical reasons, sample numbers can also be kept low. However, humans prove to be much more challenging subjects. Except in a small number of situations, factors such as diet, exercise, and medication cannot be controlled, and so a much larger number of subjects is needed in order to be able to determine a “normal” range for metabolite levels, account for inter-individual variation, and be able to detect changes above baseline.

#### 2.1.1. The Importance of Controls

It is extremely important to design an experiment containing the correct controls, in order to be able to associate observed metabolite changes with the condition being investigated [7,8]. The main types of controls to consider including are:

(a) Positive controls, where changes are expected. These can be used to check that experimental methods are working correctly, and could include a group of subjects (human or animal model) with a known disease, or a specific cell line.
(b) Negative controls, where no change is expected. These can be used to check that unknown variables are not affecting the experiments, which could result in a false-positive conclusion.
(c) Sham controls. These can be used to check effects induced by the procedure or treatment without actual use of the procedure (e.g., gastric bypass) or substance (e.g., drug).
(d) Vehicle controls. These can be used to check effects induced by a solution of the experimental compound, e.g., when a drug is administered in dimethyl sulfoxide (DMSO), the effect of DMSO on its own should be studied.
(e) Comparative controls. These act as a reference which is commonly accepted or an internal control/disease control. In cases where there is a drug treatment, it is important to test a sample of the drug to assess which (if any) signals observed in the metabolic profile arise from the drug, drug metabolites, or degradation products. Extraction blanks enable artefacts and contaminants to be assigned (e.g., from plastic tubes), and are particularly useful when extracting tissue samples.

#### 2.1.2. Confounding Factors and Variables

There are several sources of variation in metabolomics studies. Firstly, there is the biological variation in the samples themselves. Factors which can affect the metabolic profile of individuals include diet, age, medication, underlying disease, and environmental factors [9]. These are a particular issue for human subjects, as many are difficult to control, but some will be pertinent to animal models as well. When considering cell models, fluctuations in metabolite levels over time must be considered, e.g., as the cells grow and cell density changes (different cell lines grow at different rates). It is important to measure both intra- and extracellular metabolite levels to ensure that the effects observed are due to the treatment and not natural fluctuations. Also important to consider is the time of sampling, as many metabolites are subject to circadian rhythm in human and animal models, particularly hormones in blood and urine. When considering blood samples, whether the subject is in a fasting or non-fasting state should be considered, as blood glucose, amino acid, and lipid levels will be affected dramatically. There is also the variability introduced through sample collection and handling. A large body of work is available in the literature considering these factors [10,11], which is beyond the scope of this review. In summary, blood collection tubes can impact the metabolite profile due, in part, to ion suppression from anticoagulants, e.g., ethylenediaminetetraacetic acid (EDTA) [12,13]. Some serum collection tubes contain polymers such as polyethylene glycol (PEG), which is detrimental to LC-MS analysis, masking the signals from potentially important metabolites. Another consideration if collecting serum samples is the time left to clot, as metabolites such as lactate are known to change as clotting time increases, thus changing the metabolite profile [14]. When collecting urine samples, the type of preservative used, e.g., sodium azide or boric acid, may impact upon the metabolite profile [15]. The storage temperature and number of freeze–thaw cycles that the samples undergo are also important, as metabolites may degrade [16,17]. Lastly, the metabolite extraction approach (e.g., liquid extraction versus solid phase extraction; Section 2.2), extraction solvents used, and diluent also impact the metabolite profile hugely. Although some approaches may be favored over others, it is still largely subjective and will vary between research groups. The key to reproducible metabolomics data is to maintain consistency between samples as much as possible and keep the number of sampling handling steps to a minimum.

#### 2.1.3. Which Experimental Design to Choose?

There are several different experimental design types to consider. Amongst the more common are completely randomized, crossover, and factorial designs [18,19,20,21]. Although commonly used due to their fairly simple nature, completely randomized designs are limited in the fact that they study the effect of one primary factor without considering other factors. This approach would not be recommended in a metabolomics study, due to likely confounding factors (see Section 2.1.2), which may have a large impact on the metabolite profile. However, in reality, randomized studies are conducted and the confounders considered at the data analysis steps. A solution to this may be to employ a crossover design, where there could be sequential application of several treatments to the same individual (Figure 1). This means that a subject acts as its own control, thus providing smaller within-individual variation. However, the following factors need to be considered: “carryover effect”, “time-related effect”, “reversible treatment”, and “wash out period”. Factorial designs investigate the effect of more than one factor simultaneously, such as gender of the subject and response to a treatment, and so have the potential to increase information obtained from single study.

### 2.2. Sample Preparation Approaches

**Figure 2:**
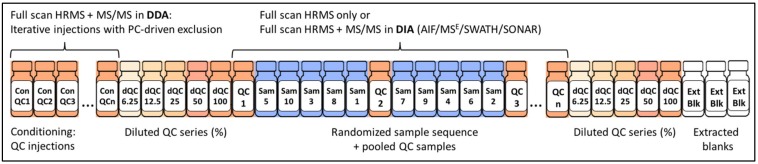
Setting up the data acquisition worklist to facilitate metabolite quantification and identification. Prior to batch run, the instrument should be conditioned (or “passivated”) using the pooled quality control (QC) of biological samples. During the conditioning, high-quality MS/MS data can be acquired in a data-dependent acquisition (DDA) mode by taking advantage of iterative injections through the application of PC-driven exclusion (of ions for which the MS/MS data have already been acquired). In this way, the amount of acquired high-quality MS/MS data will be maximized. The batch run can start (and end) with the analysis of diluted QC series that will serve to remove the features whose response is not linear; however, this removal should be performed carefully by evaluating low abundance features and those with saturation issues. Finally, samples should be run in a randomized fashion (considering the most important confounding factors, such as disease, sex, age, etc., depending on the experiment) with pooled QCs every 4–10 samples (depending on the size of the batch). Extracted blanks can be analyzed after the sample run and used for the removal of background (chemical and informatic) noise. Abbreviations: MS/MS data—fragmentation pattern, HRMS—high-resolution mass spectrometry, DDA—data-dependent acquisition, DIA—data-independent acquisition, AIF—all ion fragmentation (on Agilent or Thermo systems), MS^E^—all ion fragmentation on Waters systems^-^, SWATH—sequential window acquisition of all theoretical mass spectra or DIA strategy on Sciex systems, SONAR—scanning quadrupole DIA or DIA strategy on Waters systems.

Crucial to obtaining high quality metabolomics data is how the samples are prepared. There are many excellent papers in the literature concerning sample preparation for metabolomics studies [22,23,24,25,26,27,28,29] and individual methodologies are beyond the scope of this review. However, it is important to consider some key factors when designing the sample preparation approaches most appropriate for the biofluid or tissue of interest. These include (a) ease of method, i.e., it should be easily reproduced by different operators within the same laboratory and across laboratories; (b) there should be a minimal number of steps, so that technical/analytical variability is kept to a minimum; and (c) cost—a less expensive method will be favorable, so that it can be scaled up to larger sample numbers, such as in the case of epidemiological studies [30,31]. For untargeted metabolomics, it is desirable to use methods which do not bias for or against specific classes of analytes, so that as broad metabolite coverage as possible can be achieved [30,31]. However, it may be practical to prepare sample extracts for polar and non-polar metabolites separately, such as in the case of tissue samples [31]. In general, urine is a straightforward biofluid to prepare, as unless collected from subjects with proteinuria (or rodents), it will largely be free from protein, and so a simple centrifugation and dilution approach can be taken [30]. Be sure to ensure that the diluent used is compatible with the mode of chromatography to be subsequently employed. Plasma/serum and tissue samples require protein to be removed, which can be performed through the addition of cold organic solvent, often methanol, acetonitrile, isopropanol, or a butanol and methanol solution (BuMe), in a one-step approach [32,33,34,35]. Tissue samples require homogenization prior to protein precipitation, often using a bead beater [31]. For both blood and tissue samples, a biphasic extraction approach, such as the Bligh-Dyer or a variation (e.g., MTBE:MeOH:H_2_O), can be used [36]. Alternatively a two-step approach can be utilized, where sequential extraction of polar and non-polar metabolites is performed [37]. Particular care needs to be taken in the case of preparation of cell samples, where quenching is a crucial step in order to arrest metabolism [26,28]. It is also important to be aware of the stability of analytes, as some such as adenosine triphosphate (ATP) will degrade rapidly [26], and it may not be possible to measure these accurately. It is also important to randomize the sample preparation order, particularly in the case of large sample numbers, and to ensure that this preparation order is not the same as the analytical run order (Figure 2), so that systematic bias is minimized.

### 2.3. Data Acquisition Strategies to Facilitate Metabolite Quantification and Identification

The choice of technological platform and analytical strategy for sample analysis will be guided by the objective of the study, the metabolites of interest and the approach—untargeted or targeted—deemed most appropriate to answer the biological question. While Nuclear Magnetic Resonance (NMR) spectroscopy is endowed with high reproducibility and accuracy for metabolite measurement, MS-based technologies have made the most significant imprint in metabolomics following the introduction of electrospray ionization (ESI), which has considerably enhanced measurement sensitivity and thus promoted “omics scale” metabolite analysis [38,39]. Direct injection analytical strategies, such as flow-injection analysis (FIA), that do not apply any analyte separation have already provided an increased coverage of up to 200–300 metabolites. While this direct ionization strategy can be of particular interest in studies where high-throughput is essential, for example, in real-time metabolite profiling [40,41], it suffers from ion suppression, poor reproducibility, matrix effects, etc., allowing for only a small fraction of the polar metabolome to be putatively annotated based on accurate mass. As opposed to polar metabolites, a large body of evidence has demonstrated the value of direct infusion-based shotgun analysis for lipid identification. The latest strategies applied in shotgun lipidomics take advantage of the selective ionization of different classes of lipids in the ion source (i.e., intra-source separation under different conditions) and continuous direct injection of the sample, allowing for multi-dimensional MS analysis (i.e., multiple acquisitions in full scan and MS/MS scan modes), and thus, the unambiguous identification (including isobaric/isomeric species) and accurate quantification of lipid species (in two steps) [42,43]. Although the multi-dimensional mass spectrometry-shotgun lipidomics (MDMS-SL) improves most of the limitations related to classical shotgun lipidomics, it is relatively low-throughput and still suffers from ion-suppression, thus limiting the analysis of low abundant lipid species (unless they are derivatized) [43].

Among different hyphenated techniques, such as LC-MS, GC-MS, and CE-MS, that are complementary in their attempt to resolve chemical diversity, LC-ESI MS allows for the most comprehensive coverage of the polar metabolome and lipidome [44,45]. It allows for the simultaneous measurement of several hundred to thousands of metabolites (comprising lipids) from only minimal amounts of a biological sample in a single analysis. This coverage capacity is a benefit of LC separation that minimizes ion-suppression and maximizes measurement specificity by the separation of isobars and isomers and by providing retention time (RT) identifiers [46]. LC represents the best compromise with limited MS acquisition (scanning) speeds; by improving the specificity, and thus, S/N ratio, it enhances the quantity and the quality (i.e., purity) of acquired MS/MS data, essential for metabolite identification (in untargeted assays) and quantification (in targeted analysis) [47].

Due to inherent chemical diversity and the large size of the metabolome, there is no universal technique that can be used to assess the entire metabolome, i.e., “one size does not fit all”. The choice of LC-MS analytical strategy, including the LC and MS modes of analysis, will depend on the type of metabolites to be measured (polar vs. nonpolar) and limitations with respect to time and sample amount, which will determine how many analysis modes could be combined to expand the metabolome and/or lipidome coverage [37,48].

#### 2.3.1. LC Techniques

The most commonly used LC techniques in metabolomics include Reversed-Phase Liquid Chromatography (RPLC), ion pairing RPLC, and HILIC. Stationary phase (hydrophobic or hydrophilic), mobile phase modifiers (formic acid, acetic acid, ammonium acetate or formate, ammonium fluoride, etc.), elution gradient (from highly aqueous to highly organic and vice versa), and sample diluent will vary depending on the chromatographic mode applied. Recognized for its reproducibility and broad applicability, RPLC is predominantly used in untargeted metabolomic assays. While RPLC can be used for profiling of mid-polar and non-polar metabolites, including complex lipids, recently, the major challenge in metabolomics has been the separation of highly hydrophilic central carbon metabolites [49], specifically to understand the metabolic shifts in cellular metabolism under different conditions. To enhance the poor retention of hydrophilic metabolites by RPLC, ion pairing agents (e.g., alkyl sulfonates or heptafluorobutyric acid in positive mode, and long chain tertiary/quaternary amines such as tributylamine in negative mode) can be added into the mobile phase, where they combine with the analyte (i.e., cations or anions) to form an ion pair that can be efficiently retained by the reversed phase packing [50]. Yet, this strategy is not MS friendly, with the background signal of ion pairing agent causing system contamination and resulting in notable ion suppression and reduced sensitivity, thus demanding a dedicated LC-MS system. Alternative strategies, such as multimode C18 columns that contain cation and anion ligands (e.g., HSS T3 Waters, Milford, MA, US, Scherzo SM-C18 Imtakt USA) and, in particular, HILIC, have been developed and have become increasingly robust and popular for polar compound retention [51,52]. Indeed, stationary phases with derivatized silica, including diol, amine, and amide, have proven their efficiency and robustness in the separation of polar molecules through multiple mechanisms, such as partitioning between the mobile phase and enriched water layer on the stationary phase, hydrogen bonding, dipole–dipole interactions, etc. In addition, the stationary phases with zwitterionic functional groups (with the polymeric support, e.g., ZIC-HILIC and ZIC-pHILIC, ZIC stands for zwitterionic stationary phase) offer excellent performance in the retention of highly polar metabolites (e.g., di- and tri-carboxylic acids, phosphorylated energy currency metabolites) via ion exchange, and wide pH range stability (from 2 to 10) [51,53]. Besides polar metabolite separation, HILIC has also been increasingly used for complex lipid separation by class, according to polar head groups [54,55].

For an untargeted metabolomics experiment, one would ideally maximize data acquisition and metabolome coverage by combining HILIC and RPLC in both positive and negative ionization modes. Analysis using HILIC in acidic conditions in positive ionization mode would allow for the assessment of amino acid and acylcarnitine metabolism [56], while the analysis in basic conditions in negative ionization mode would provide insight into glycolysis, tricarboxylic acid cycle (TCA) cycle, purine and pyrimidine metabolism, etc [51]. Analysis using RPLC and non-polar eluents (often a combination of isopropanol (IPA) and acetonitrile would allow for comprehensive lipid profiling, including glycerolipids (TAGs—triacylglycerols, DAGs—diacylglycerols, and MAGs—monoacylglycerols), cholesterol esters (CEs), sphingolipids (sphingomyelins, ceramides), glycerophospholipids (PCs—phosphatidylcholines, PEs—phosphatidylethanolamines, PSs—phosphatidylserines, PIs—phosphatidylinositols, PGs—phosphatidylglycerols), and free fatty acids [57]. These analyses can be performed following two-phase extraction (e.g., MTBE/MeOH/H_2_O) or single step extraction using isopropanol or butanol and methanol solution (BuMe). When time and sample amount are limited, the researcher should decide depending on which metabolite classes are of the utmost relevance to answer the specific biological question.

#### 2.3.2. Mass Spectrometry Acquisition Modes

Following LC separation, MS detection must be performed in optimized conditions to acquire maximal high-quality MS and MS/MS data for metabolite quantification and identification (Figure 2). Optimal MS acquisition conditions are instrumentation-dependent and comprise ion source and analyzer parameters. For an untargeted experiment, data are usually acquired in full scan mode, where the instrument is set to scan the complete mass range from 50 to 1200 Da. Despite the fact that increasing mass-resolving power is beneficial to resolve co-eluting isobaric compounds and we may say that “*the higher the resolution the better*, *there may never be enough resolution to separate all the metabolites present in complex biological matrices*”, in the small molecule “world”, many compounds have the exact same accurate mass [58]. From this point of view, resolution becomes less important when compared to instrument scanning speed and sensitivity, essential for acquisition of maximum high-quality MS/MS data necessary to translate putative hits into metabolite identities [47,53]. During sample analysis, HRMS data acquisition can be followed by sequential acquisition of MS/MS data using data-independent acquisition (DIA; such as all-ion-fragmentation (AIF) in Agilent Q-TOF, MS^E^ in Waters Q-TOF, or SWATH in Sciex TripleTOF, and BASIC DIA in Orbitrap) with a minimal loss of sensitivity (approximately two times), or MS/MS data can be acquired only on pooled QC samples at the end of the run, in DIA or in data-dependent acquisition mode (DDA with a focus on top “n” ions, Table 1). In data-independent acquisition (DIA), all fragment ions for all precursors are acquired simultaneously, while in data-dependent acquisition (DDA) the ions for MS/MS acquisition are selected in real-time based on threshold intensity [59]. Finally, the filtered metabolite features of interest (i.e., those that vary significantly between two or more analyzed conditions) can be targeted for MS/MS data acquisition in selective or targeted MS/MS mode, a posteriori, following data processing, filtering, and statistical analysis. The pitfall of this strategy is the time lapse (and thus possible sample alterations) between the first batch of analyses in MS mode only, for relative quantification, and the targeted run to acquire MS/MS data on ions of interest for their identification.

Although DDA is still the most popular simultaneous MS/MS acquisition mode used, DIA is gaining attention following the development of MS/MS data deconvolution algorithms (to link precursor and product ions) and improved coverage for low abundant precursor ions [60,61,62]. In general, the quality and the amount of acquired MS/MS data depend on instrument acquisition speed and sensitivity (also related to metabolite ionization efficiency). With regards to instrument scanning speed, in DDA, attention should be paid to the *m/z* resolution window (wide vs. medium vs. narrow), the accumulation times, and the number of targeted precursor ions per scan [47]. To avoid the selection of the most highly abundant ions each time, across multiple scans, a preferred list of ions of interest can be defined and contaminant ions placed on the exclusion list. Data can also be acquired in a time-staggered fashion through a set of iterative injections (of pooled QC samples) with PC-driven exclusion (of ions on which the data has already been acquired in previous runs), which will significantly enhance the amount of acquired MS/MS data [63,64].

**Figure 3:**
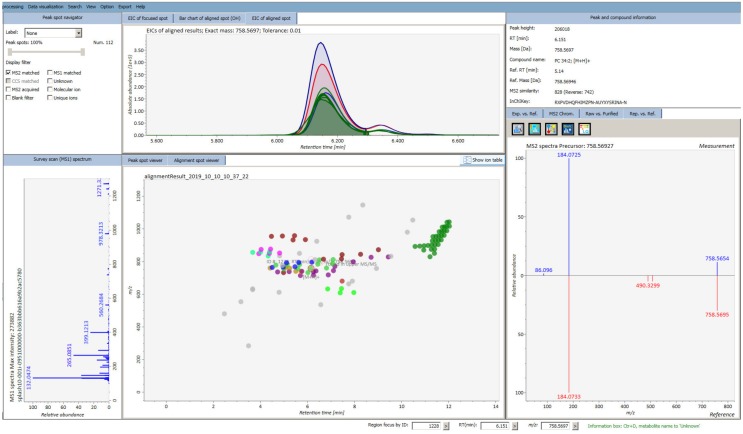
Overview of lipidomic data analysis (acquired by DDA) using MS-DIAL, the open-access software designed for simultaneous metabolite quantification and identification. Displayed are the MS/MS matched peaks (each lipid class is differently colored) with the example of phosphatidylcholine annotation using MS/MS matching against LipidBlast.

DIA can be applied in an all-ion-fragmentation mode (AIF, MS^E^) where the first quadrupole (Q1) transmits the full mass range (*m/z* 50–1700) of precursor ions in the collision cell, or with sequential windows (SWATH and SONAR on Q-TOFs or BASIC-DIA on Orbitrap), where the Q1 will transmit several increments (20–50 amu) across the mass range of interest sequentially in the collision cell (Table 1) [59]. Here, again, the number and the size of mass windows will depend on the instrument acquisition speed. A major challenge related to DIA is to re-establish the direct link between the precursors and their fragment ions or to correctly deconvolute the MS/MS spectra. The wider the isolation window for precursor ion selection, the higher the contamination is of MS/MS spectra, making their deconvolution more difficult. Several algorithms have already been successfully implemented proving their efficiency in MS/MS data deconvolution (MS-DIAL [62], MetDIA [65], DecoMetDIA [66]), with a major limitation being the comprehensiveness of experimentally acquired spectral databases (e.g., METLIN [67], NIST, MoNA, MassBank [68], mzCloud, GNPS [69], etc.). Due to time-consuming standard characterization to expand these experimentally-derived spectral databases, considerable efforts were put towards the development of computational tools for in silico generation of mass spectra used for MS/MS data matching and metabolite annotation (e.g., iMet [70], LipidBlast [71], MetFrag [72], MetDNA [60], CSI:FingerID coupled to Sirius [73]; see section on metabolite identification below; Figure 3).

It should be emphasized here that coupling of ion mobility (IM) analyzers, as an additional separation technique to conventional LC-MS/MS analysis, can markedly facilitate metabolite identification, and even resolve stereoisomers [74]. The separation of ions according to their size and conformation prior to MS/MS data acquisition will also enhance spectral clarity and fragmentation specificity. Importantly, experimental collision cross-section (CCS) values can be computed (using drift tube ion mobility MS or DTIMS and traveling wave ion mobility MS or TWIMS) with very high reproducibility (Relative standard deviation or RSD < 2%) [75]. FAIMS (high-field asymmetric waveform ion mobility MS) is atmospheric pressure IM technology that can also be used as an orthogonal separation approach (also known as DMS or differential mobility spectrometry), although it does not allow for the acquisition of CCS values.

In an untargeted metabolomics experiment, one would ideally acquire as many MS and MS/MS data as possible, simultaneously, or at least within the same analysis batch. This would allow for the simultaneous metabolite quantification and identification (via MS/MS matching against spectral libraries and using computational tools like Sirius [73]) in an automated fashion. To reach optimal metabolome coverage and annotation, there is room for improvement on the instrumentation side (i.e., limited acquisition speeds, and sensitivity related to ionization efficiency and ion transmission), the need to enhance the comprehensiveness of spectral libraries (taking into consideration the exposome), and to improve the computational approaches for annotation of unknown metabolites.

It is worth noting that the fastidious metabolite identification process in untargeted experiments often yields the identification of “*known (un)knowns*”, as a consequence of the above-specified remaining challenges. This bias encouraged the development of high-coverage targeted methods for quantification of polar metabolites and lipids to bridge the gap between untargeted and targeted approaches. These methods can be strategically derived from DIA methods, such as SWATH, capable of acquiring MS/MS data for all detectable metabolites in a biological sample [76]. As a library of Multiple Reaction Monitoring (MRM) transitions, acquired on different instruments, the METLIN-MRM can be particularly useful to accelerate the development of broad-scale MRM methods [77].

### 2.4. Data (Pre)Processing: from Peak Detection to Profile Alignment

#### 2.4.1. Software for Data Pre-processing

The amount of raw data generated from an untargeted metabolomics study using mass spectrometry is often huge, with large file sizes (possibly up to 1–2 GB per sample) depending on the instrumentation used. Therefore, there is a need for large computational power or use of computational clusters or clouds for data processing. The data pre-processing pipeline consists of several important steps in order to extract the maximum useful information from the data, whilst eliminating redundancy. The many different software available for performing these data pre-processing steps range from MS vendor software to freely available scripts and software. Some examples of freeware are XCMS [78,79], MZmine2 [80], and MSDial [62] There is also the XCMS online platform [81,82], where you can upload your data and the processing will be performed for you, employing parameters set within the software.

#### 2.4.2. Important Steps in Data Pre-Processing

The first, crucial step is peak detection (or extraction). At this stage, the files are uploaded (read) into the software, and using a selected algorithm, the software will search for any peaks in the samples. A peak (or metabolite feature) may be defined as a distinct ion species with a unique *m/z* ratio and retention time (RT). It is important to note that one metabolite can be represented by multiple peaks or distinct ion species, namely, isotopes, adducts, in-source fragments, or multiple charged species. This peak detection is normally split into two steps: (1) Separation of mass traces and (2) filtering or detection of chromatographic features. The parameter settings at this stage will be important, such as signal to noise ratio (S/N) and width of the chromatographic peak, in order to enable the detection of peaks with very low S/N ratios while simultaneously filtering out random noise. These parameters, as well as maximal *m/z* deviation, can be calculated by looking at the raw data files of QC samples across the analytical run or similar (e.g., selected study samples across the run) and specified in the pre-processing parameters. Peak width range should be calculated using the narrowest and widest peaks in the chromatograms, again determined visually from QC samples or similar. Extracted ion chromatograms can be constructed to aid determination of these parameters. Similarly, S/N and *m/z* deviation should be calculated across the elution profile using high and low intensity peaks to ensure an accurate calculation. Typically *m/z* deviation is ~5 ppm for Orbitrap data and ~25 ppm for Q-ToF data. Once these peaks have been extracted, they need to be grouped, or matched, across all the samples in the dataset. This is to enable peak areas (or, in some cases, peak heights) to be compared across the samples in a semi-quantitative fashion. Untargeted metabolomics experiments can be large, particularly in the case of epidemiological studies where thousands of samples may be analyzed in a single run or across batches. Usually, retention time alignment is needed, as there may be peak shifting across the analytical run (due to changes in pH or temperature, column aging or build-up on the column). However, this is less frequent since the advent of U(H)PLC, and the authors have found that in the case of small datasets, retention time correction may no longer be required. Nonetheless, it is important to assess each dataset individually, and as most software performs this retention time alignment, it is generally advisable to do so. The output at this stage will be a peak table containing *m/z*, RT, and abundance for each metabolite feature (peak) in every sample [83,84,85]. Depending on the software employed, grouping of isotopes/adducts, etc., may have been performed—if it has not, then software such as CAMERA, AStream, RAMClust, and the recently developed METLIN In-source fragment Annotation (MISA) [86,87] exist within the R environment to assist with this grouping and, therefore, data reduction [88,89,90]. Further, peak annotation may have been performed in some instances through linking with databases, such as with XCMSOnline. This peak table can then be further analyzed, either within the same software or using dedicated software such as SIMCA (Umetrics). Freeware available includes Metaboanalyst [91], a multipurpose software which can also provide pathway analysis tools.

#### 2.4.3. Dealing with Artefacts

The output from the data pre-processing software can be very large and complex, depending on the peak picking parameters, as described in the previous section. As instrument sensitivity increases, so does the likelihood of picking up noise and artefacts in the data. Artefacts can include solvent clusters, contaminants (from the column, vials, or solvents), and other spurious signals. These inflate the data and so need to be removed; thus, there are several approaches to tackling this challenge. A widely used approach in the metabolomics community since 2006 is the employment of QC samples [92]. These generally take the form of a pooled samples comprised of aliquots of all study samples, but may be a subset of samples if the size of the study is large [93]. Occasionally, a “surrogate” QC sample could be used, such as the NIST reference plasma material [94].

#### 2.4.4. The Importance of Quality Control

QC samples play a crucial role in untargeted metabolomics studies, in terms of monitoring system stability and data quality (summarized in Table 2). The QC sample will be injected at the start of the analytical batch in order to condition the column and assess instrument stability; the number of injections required may be sample- and column-dependent, but is often in the region of 10 injections [95,96]. Then, the same QC sample can be injected every 4–10 samples, making up to ~10% of the sample injections. This within-run QC can be used to assess stability within the run, e.g., retention time and signal intensity drifts. Importantly, a QC dilution series can be employed; this takes the form of serial dilutions from the QC sample [93]. The purpose of this dilution series is to identify and remove peaks (metabolite features) that do not respond to dilution in a linear manner (as determined by calculating coefficient of determination (r^2^ or R^2^) values), as they are likely to be noise, or at least non-biological in origin. Additionally, the coefficient of variation (CV) can be calculated for every metabolite feature in the within-run QC samples. Features with a CV above a certain threshold, e.g., > 30%) can be removed from the dataset, as they are unlikely to be reliable biomarkers [30]. In some cases, metabolite features which appear in below a certain proportion of the QC samples (e.g., in < 75% of samples) could also be removed from the data. Lastly, the analysis of blank samples, such as blank mobile phases and also extraction blanks (where the sample preparation procedure has been followed but in the absence of biological sample), can provide valuable insight into the origin of many of the metabolite features reported. Those that appear in the blank samples are again likely to be non-biological in origin and so can be removed from further processing steps [93]. These data filtering and reduction steps can dramatically reduce the size of the dataset and streamline the subsequent data analysis procedure.

### 2.5. Univariate and Multivariate Statistical Data Analysis

Untargeted metabolomics studies generate a wealth of data, from which meaningful biological interpretations are desired. Statistical analysis of the data is another hugely important step in the metabolomics pipeline; therefore, there are many important parameters which must be considered. The most typical workflow is to perform multivariate analysis followed by univariate analysis in order to elucidate and validate potentially discriminatory metabolites [98,99].

#### 2.5.1. Multivariate Approaches

Multivariate analysis encompasses methods to reduce the complexity of data, such as that generated from a metabolomics study, where the number of variables (in this case, metabolite features) is greater than the number of samples. Multivariate analysis can be performed using vendor software, programming platforms such as R and Metaboanalyst, or commercial software such as MATLAB^®^ (MathWorks) or SIMCA (Umetrics).

#### 2.5.2. Principal Components Analysis

**Figure 4:**
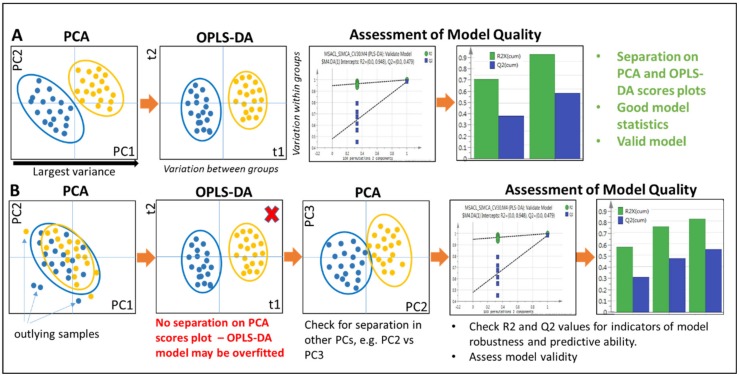
Simplified overview of PCA and OPLS-DA showing (**A**) good separation on PCA and OPLS-DA scores plots. High R2 and Q2 values indicate good model robustness and predictive capability. Permutation test indicates a valid model. (**B**) No separation on the PCA scores plot of PC1 vs. PC2, but separation is still achieved using OPLS-DA. In this instance, the model could be overfitted and unreliable. It is advisable to check for separation in other components, e.g, PC2 vs. PC3, as well as to assess R2 and Q2 and perform permutation tests. CV-ANOVA can also be used to assess model validity (not shown).

The first step is generally an unsupervised approach, such as principal components analysis (PCA), which can be used to visualize data structure, class differences, and outliers (Figure 4). PCA can be considered as to be finding *maximal variation* between the groups of interest. Importantly with unsupervised approaches, no class information is given, and so an unbiased view of class separation can be obtained. When visualizing a PCA scores plot, the first principal component (PC1) explains the largest variation in the data, followed by PC2, PC3, etc. Multiple classes can be viewed on the scores plot, in two or three dimensions, and so group separation can be observed, e.g., over time. The loadings plot provides an indication of which metabolite features are responsible for any observed separation, e.g., between classes, and can be mapped onto the scores plot if desired, in what is known as a bi-plot.

#### 2.5.3. Supervised Approaches

Once separation has been assessed, supervised analyses can be performed, such as partial least squares discriminant analysis (PLS-DA) and its orthogonal counterpart, OPLS-DA. These approaches incorporate class information and so find a way to achieve the maximal separation between the classes of interest. In the scores plots, the x-axis shows the variation *between* the groups, while the y-axis shows variation *within* the groups. These methods can suffer from the risk of over-fitting the data—they can produce class separation even with random data—and must be interpreted with caution (Figure 4) [100]. This can have detrimental downstream impacts on biomarker discovery and validation as results may not be reliable or reproducible. R2 and Q2 values can be used to assess the model robustness and predictive power; these values will be low—particularly the Q2—in an overfitted model. A low Q2 indicates that new data would not be predicted accurately in the model. Further, machine learning-based model validation approaches, such as CV-ANOVA (based on ANOVA of the cross-validated residuals), can assess model validity [101]. Permutation tests can also be used to assess the significance of a classification. The class assignment is permuted repeatedly, with a model between the data and the permuted class-assignment built for each permutation. These models are then compared with the original multivariate model [102]. Variable Importance for the Projection (VIP) scores can be used to identify the metabolite features contributing most to any class separation; VIP scores > 1 are suggested to be important, whilst those < 1 are suggested to be unimportant for the model. The range of VIP scores will vary with each dataset and, in some studies, there may be hundreds of metabolite features with a VIP score around 1, meaning that the cut-off applied is much higher. OPLS-DA S plots can also be used to identify discriminatory metabolite features warranting further investigation.

#### 2.5.4. Univariate Methods

Even though multivariate analysis tools can be useful for exploring metabolomics data and guiding researchers towards potential discriminatory biomarkers, there are several pitfalls to these approaches. As discussed above, supervised models suffer from the risk of overfitting. Datasets containing a large amount of sparse data (in terms of the number of input variables) or missing data (which can occur with some pre-processing tools) may compromise model performance [103]. To this end, features which have been proposed as discriminatory from multivariate analyses can be further validated using appropriate univariate statistics [81]. However, univariate tools are also not without their challenges, and it is easy to inadvertently apply the wrong statistical test to a dataset. It is important to assess the data at the start to ensure the correct test is being performed, e.g., whether to use a parametric or non-parametric test. A rule of thumb is that if the data are normally distributed, then a parametric test, such as a *t*-test, can be used. Normality can be tested using, e.g., the Shapiro–Wilk test, which is good when the sample size is < 50. Note that it is not possible to assess normality of the distribution if the sample size is small, and some tests do not cope well with small sample sizes. Parametric tests are considered to be more powerful than non-parametric tests, with less risk of a false negative (i.e., non-significant) result than with a non-parametric test. However, when dealing with populations that are non-normally distributed, with unequal variances and/or unequal small sample sizes—all possible in untargeted metabolomics—often a non-parametric test can perform better [81]. An additional complication is that univariate tests applied separately to numerous variables will overlook correlations within metabolite features, which may be important in elucidating related metabolites and interpreting biological pathways.

#### 2.5.5. Multiple Comparison Testing

In untargeted metabolomics studies, it is likely that the number of metabolite features (variables) is greater than the number of samples analyzed [104,105]. If univariate tests were performed on each of these variables, the false discovery rate (the chance of significance being found) is high. These are known as Type I errors (false positives) and must be addressed if valid metabolite markers and meaningful biological conclusions are to be found. To combat this issue, multiple comparison testing can be performed. Commonly used approaches for false discovery rate correction (FDR) are the Bonferroni correction (a conservative method) or the less conservative Benjamini–Hochberg or Benjamini–Yukatelli corrections. These will adjust the *p*-value cut-off, meaning that fewer variables will reach significance and, therefore, there will be fewer false positive results. Using a combination of multivariate and univariate testing, a potential biomarker should have a VIP > 1 and a *p*-value < 0.05 (or the corrected value after FDR—false discovery rate correction) [106].

### 2.6. Metabolite Identification: From Spectral Database Matching to Computational Approaches for Unknown Metabolite Annotation

Following feature filtering using QC-based estimates (see Table 2) and statistical criteria to extract the metabolite features of interest, the next challenge constitutes assigning the identity to these features and placing them in a biochemically relevant context for data interpretation. As specified in Section 3, LC-MS is not only the most versatile and comprehensive methodology with respect to metabolome and lipidome coverage, but also provides important information for metabolite structure elucidation, including RT, accurate mass, isotope distribution, and MS/MS fragmentation pattern, in addition to IMS (and/or CCS value). Despite this, the majority of metabolite features in untargeted metabolomic datasets (approximately 80%, so-called “dark matter”) remain un-annotated or misidentified [75,107,108], hiding many unknown metabolites, but also high levels of chemical and informatic noise (artefacts of peak detection algorithms) and redundancy (due to defects in feature annotation and grouping algorithms). We distinguish two main bottlenecks, one associated with known metabolite misidentification and another one related to unknown or novel metabolite identification (see Table 3).

Metabolite identification starts, in general, by database searching using accurate mass (*m/z*) measurements (up to 4 decimal places) and prediction of elemental composition (i.e., molecular formula). Accurate mass searches yield many putative hits, including potentially false matches due to the presence of isomers, interferences between the metabolites of similar molecular weight (i.e., isobars), and mis-annotation of in-source fragments and even certain adducts (see Table 3) [26]. In most cases, the MS/MS fragmentation pattern, defined by the product ion masses and their relative abundances, will provide sufficiently specific data to confirm the metabolite identity with a high level of confidence. The exceptions are structural and/or stereoisomers (i.e., L- and D-serine, for example, or complex lipids differing only in positions of unsaturations), which can be distinguished only with the additional chromatographic resolution (RT, chiral columns) and/or IMS (and CCS values) data.

MS/MS spectra acquired from samples will be matched against spectral databases containing experimentally acquired spectra on pure standards (e.g., METLIN [67,109], NIST, mzCloud) or any annotated structures (community databases such as MassBank [68], including European MassBank, MassBank of North America, and GNPS based on crowd sourcing [69]). The content of these databases has been extensively reviewed in several recent publications [75,110]. MS/MS spectra matching is usually followed by the similarity score calculations for matches (e.g., METLIN online database) and ranking of candidates based on the similarity to the reference spectra [47,111]. While five different levels of reporting confidence in metabolite identification have been established by the Metabolomics Standards Initiative [75], absolute identity can only be made when an authentic commercially available standard has been compared to the analyte of interest and found to match all applicable measurements (accurate *m/z*, MS/MS, RT, etc.). When standards are not available, the unknown metabolite of interest needs to be isolated from the biological matrix (e.g., plant, fungi, sponge extract) using LC, and the combined LC-HRMS and NMR analysis will allow for structural elucidation. The novel metabolite identity needs to be confirmed by custom synthesis of standard and its analysis under the same analytical conditions.

To facilitate and automatize metabolite identification, significant efforts were made to further expand the experimentally-derived spectral libraries by MS/MS data acquisition (on different instruments, collision energies, and ionization modes) and sharing. However, compared to the size and diversity of endogenous and exogenous metabolome, this conventional method of metabolite annotation by matching the experimentally acquired MS/MS spectra to standard spectral databases remains limited by the size of databases and the lack of commercially available standards for many cellular metabolites. To address this problem, recently, the computational metabolomics community has grown to develop and improve computational approaches for known and unknown metabolite identification (Table 3). These computational metabolomic approaches employ two main strategies: (1) In silico prediction of fragmentation MS/MS spectra from chemical structures of known compounds, and (2) in silico prediction of molecular substructures (i.e., molecular fingerprints or feature vectors that encode the structure of a molecule) and general chemical properties of the unknowns from experimentally acquired MS/MS spectra [112]. With the in silico fragmentation methods, the experimentally acquired spectra of an unknown metabolite (for which reference spectra are not available) can be matched against in silico theoretically predicted spectra simulated on known candidate structures retrieved from databases (Human Metabolome Database (HMDB), PubChem, KEGG, etc.) [113]. In silico fragmentation from chemical structures of known compounds can be computed by rule- (e.g., MS-FINDER, LipidBlast), combinatorial- (e.g., MetFrag), and machine learning-based methods (e.g., CFM-ID) [75]. Rule-based generation of specific fragmentation patterns and heuristic modeling of ion abundances is efficient for classes that have consistent and predicative fragmentation patterns, such as lipids (e.g., LipidBlast).

The in silico prediction of molecular substructures are machine learning-based methods that can translate the MS/MS spectra to metabolite structure information. To learn the mapping of an MS/MS spectrum to a molecule structure, these methods need to be trained on spectral databases of known metabolites. In general, machine learning methods can be divided in two groups, supervised learning for substructure prediction (e.g., CSI:FingerID) and unsupervised learning for substructure annotation and grouping of metabolites based on shared, biochemically relevant substructures (e.g., MS2LDA) [112,114,115,116]. The main objective of supervised methods, such as CSI:FingerID integrated in Sirius tool, is to determine, using a database of molecular structures, the structure that best fits the experimental data. In Sirius 4, the assessment of molecular structures from MS/MS data can be performed automatically for the entire LC-MS dataset (rather than per spectrum) and MS data-driven annotations can be obtained for all detected features [73]. These machine learning approaches were essential for the recent progress in metabolite identification and will pave the future of metabolite structural identification.

Data sharing will also be key to advance these computational approaches. There are two main repositories that can be used for metabolomics data sharing, the Metabolomics Workbench (US, [117]) and MetaboLights (EU, [118]). There is space for the improvement of data upload, which demands fastidious data preparation due to considerable requirements on sample and method related metadata.

### 2.7. Metabolite Features and/or Metabolites to Pathways and Metabolic Networks

#### 2.7.1. Metabolic Networking for Metabolite Identification

While pathway and network analysis are mainly used to facilitate metabolite data visualization and interpretation, the biochemical knowledge about chemical reactions (i.e., metabolite conversions via enzymes) and metabolic pathways integrated within a metabolic network (to sustain cellular function) can also be used to facilitate metabolite identification. As an alternative to the above-described tools relying only on the spectral data and information related to molecular (chemical) structure, several approaches, such as Mummichog [119], PIUMet [120], and MetDNA [120], based on the “*features to pathways*” principle, have been developed to facilitate and speed up metabolite identification using reference metabolic network models. This biochemically relevant information can guide with respect to the metabolites that the organism of interest is able to produce and thus increase the confidence of metabolite annotations (see Table 3) [121]. Both Mummichog and PIUMet rely on the assumptions that locally enriched metabolite matches within the metabolic network are true, while false matches will distribute randomly. Both tools will infer metabolically active pathways without requiring metabolite identification. Finally, metabolite identities will be predicted and chemical information on annotated isotopes and adducts will be used to evaluate the prediction confidence level. Metabolite annotation and Dysregulated Network Analysis, or MetDNA, uses the metabolic network knowledge for the annotation of known metabolites (from highly conserved primary metabolism) detected in untargeted experiments. Annotation starts from the set of identified “seed” metabolites by predicting their reaction-paired neighbor metabolites on the assumption of their structural similarities. Through the reiterated application of this recursive algorithm, the number of annotated metabolites will be progressively propagated and significantly enhanced (to up to 2000 metabolites from one untargeted experiment) [60]. Using a similar principle, the GNPS or Global Natural Products Social Molecular Networking will construct the molecular similarity network based on the similarity of MS/MS spectra (two metabolites share similar MS/MS data due to their structural similarity) with the aim to annotate the unknown natural products using already annotated metabolites (by the community) within the same sub-network. While these networking approaches are fast and valuable for the reduction of metabolomic datasets, however, annotation remains ambiguous and should be validated through more specific targeted MS/MS analysis.

#### 2.7.2. Metabolic Networking to Visualize and Interpret Metabolite Changes

In general, changes at the metabolite level cannot be looked at independently outside of the context (of their interactions with other metabolites, proteins, and genes), and meaningful changes can be missed by relying only on the arbitrary significance threshold (or *p*-value). It is thus of the utmost importance to interpret identified alterations at the metabolite level within the metabolic networks, especially when it comes to the discovery and understanding of subtle (fold change < 2) but coordinated and physiologically relevant changes, often the case in biomedical and human population studies. Metabolic networks, derived from genome-scale metabolic network models (GSMNM) are the most accurate ways to describe and represent metabolism, as compared to discrete pathways [122]. Multiple metabolic pathways share metabolites, and the synthesis of one metabolite can require the integrated cooperation of more than one pathway. The reconstructed GSMNM from annotated gene–protein reaction (GPR) associations can define the metabolic capacity of a model organism(s), in any specified condition. While the primary metabolic pathways are highly conserved across model organisms, they can be differentially regulated, in an organism-specific manner, as a function of genetic effects (i.e., mutations in different genotypes) and environmental exposures. Efforts are needed for systematic characterization of the model organism metabolomes (across different conditions, using quantitative information), and to develop compartmentalized models for different organs and host–microbiome metabolic interactions [123,124,125,126].

To interpret data from metabolomics experiments and gather biologically meaningful information, one would ideally perform two types of analysis: (1) Mapping and visualization of metabolite changes in the graphical representation of cell metabolism, i.e., metabolic network; and (2) statistical analysis to determine the overrepresented pathways, known as metabolite set enrichment analysis (MSEA). Most of the open access tools designed for pathway and network analysis provide both of these functionalities, visualization to assess if metabolites are involved in the same pathways and how they are connected within a metabolic network and enrichment analysis to highlight the pathways associated with the examined phenotype. The open access software that provide these functionalities in the interactive fashion are listed in Table 4. For the computational community, the recently assembled MetaRbolomics toolbox provides an extensive resume of R packages that can be used for data processing, metabolite annotation, and biochemical network and pathway analysis [127].

In order to map the identified metabolite changes in the biochemically relevant context, one first needs to convert the metabolite identities into the relevant metabolite identifiers (e.g., KEGG, HMDB, Recon, etc.) that can be used for mapping to metabolic networks derived from genome-scale models (as a product of genome sequencing, annotation, and, finally, metabolic model reconstruction). The conversion to different metabolite identifiers can be executed in batches using a chemical translation service, provided by UC Davis [134] or MetaboAnalyst [91]. Users should consider that a portion of identifiers may be missing and/or incorrectly matched (approximately 10%), thus manual curation may be necessary prior to the upload to pathway or network analysis tools for further analysis and visualization. Metabolite mapping would ideally be based on InChIs or InChIKeys, requiring that these identifiers are specified in both databases and networks [126]. There is an important challenge here regarding lipids, due to the ambiguous identification given by sum composition (i.e., PC 34:2) that can correspond to many similar lipid species having different fatty acid composition (16:1/18:1, 16:0/18:2, etc.) [132].

**Figure 5:**
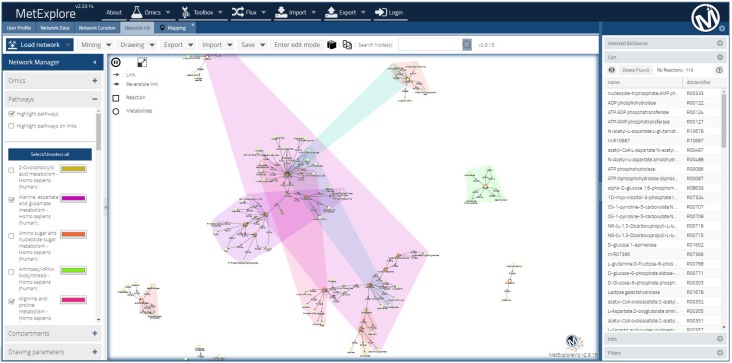
Metabolite mapping on the metabolic networks—an overview of MetExplore network Viz functionalities. The projected network has been created from the list of chemical reactions (in the cart on the right side of the figure)—derived from the list of identified metabolites whose levels varied significantly (as a result of brain cell profiling). The extent of each pathway has been encircled and colored for visualization. Alanine, aspartate and glutamate metabolism, and arginine biosynthesis have been highlighted as enriched (using integrated ORA).

Visualization of metabolite changes in the context of metabolic networks brings together chemical reactions (of which metabolites are the products or substrates), and the genes coding for the enzymes making these reactions possible. MetExplore is among the most comprehensive tools that allows for the construction of tailored networks and collaborative curation and annotation of metabolic models, in addition to the interactive network visualization, from the entire network down to detailed sub-networks (build from selected network elements—a pathway or a set of genes) [128,135]. In addition to visual inspection, flux consistency is checked for the metabolic model, i.e., network, validation. MetExplore integrates a large panel of metabolic models (called “biosources” in MetExplore) depending on the model organism, and each metabolic network can be exported as an SBML or Excel file. Mapping of metabolites can be achieved using their network identifiers (KEGG, Recon, etc.) and, further on, smart filters can be applied to select the reactions involved in a combination of pathways (e.g., enriched pathways) of interest to be visualized—through the MetExploreViz web module (Figure 5). The MSEA is integrated and performed using hypergeometric tests (corrected with Bonferroni or Benjamini–Hochberg methods). Specific metabolites and pathways can be highlighted, edited, and exported, and the shortest paths between the metabolites of interest can be automatically extracted to reduce visual complexity, thus allowing for data mining.

As mentioned above, in addition to metabolic (sub)network visualization, metabolite set enrichment analysis (MSEA) as a metabolomic counterpart of the gene set enrichment analysis (GSEA) and/or over-representation analysis (ORA) are used to investigate the metabolic pathways whose activity differs among analyzed conditions (e.g., CTRL vs. disease). MSEA takes into consideration the quantitative measure associated with each metabolite (i.e., abundance or concentration, and fold change) [121]. MSEA firstly assigns metabolites to pre-defined groups of functionally related metabolites (or metabolite sets) based on references databases (e.g., KEGG, HMDB; Table 5). The metabolite sets can be defined as biochemical or signaling pathways (i.e., metabolites involved in the same biological process), pathways associated with a metabolic disease (i.e., metabolites that vary significantly under the same pathological conditions, suggested by HMDB, [136,137]), pathways active in specific organs, tissues, or organelles (i.e., metabolites present in the same location, suggested by HMDB, [137]), etc. MSEA then applies *Globaltest* [138] to detect the subtle but consistent and coordinated changes (i.e., differences) among the group of metabolites (i.e., pathway) between two conditions, and thus identifies the affected (or deregulated) biochemical pathway associated with the analyzed outcome or phenotype [91,121]. The obtained *p*-value gives the probability that none of the matched compounds in the group of metabolites is associated with the phenotype. A closely related approach to MSEA, an over-representation analysis (ORA), is used to evaluate the probability that the particular set of metabolites (e.g., biochemical pathway) is represented, within a defined list of metabolites of interest, more than expected by random chance. For ORA, a user can provide only a list of metabolite identifiers, corresponding to metabolites that vary significantly between two analyzed conditions. Several probability tests, such as Fisher’s exact test, binomial probability, or hypergeometric distribution test, can be applied, followed by the correction for multiple testing. Here, the reference metabolome should comprise the metabolite sets that can be detected in the analytical conditions used, thus reflecting the analytical method coverage. If the entire library of metabolite sets is used for ORA by default, the observed enrichment may be a consequence of applied analytical platform bias instead of being biologically relevant. ORA and/or MSEA are integrated in many different pathway and/or metabolic network analysis software, such as MetaboAnalyst, MetExplore, Pathvisio, etc. Finally, MetaboAnalyst allows also for the combined MSEA and pathway topology analysis that will display pathway impact values based on centrality measure—local quantitative measure of the position of a node (or a «key» position) relative to the other nodes in the network.

Although the tools for metabolic network analysis are being steadily improved by the computational community, there are still a number of challenges, related to metabolome coverage bias of the experiment (i.e., analytical limitations), scarcity of well-annotated metabolomics data (number of unknowns or non-annotated metabolite(s) (features) remains high), and, finally, the lack of knowledge about network regulation. It is also important to consider that the metabolome cannot be computed directly from the genome, and that many metabolites still need to be integrated into our current metabolic networks, thus making use of the wealth of data generated in metabolomic experiments.

### 2.8. From Untargeted to Targeted Assays

Global or untargeted metabolomics provides the opportunity for biomarker discovery and hypothesis generation. Potentially, it can enable the elucidation of the involvement of previously unknown or unsuspected pathways in disease states or in response to therapy. Inherently, this untargeted approach does not bias for or against specific analyte classes and provides a wide view of the metabolome. Sample preparation and analytical methods are somewhat generic and are usually optimized for sample type. However, with this approach comes the bottleneck of metabolite feature annotation and metabolite identification, as described in this review. Therefore, high-coverage targeted assays are becoming more prominent in the field of metabolomics. With targeted assays, tandem or triple quadrupole mass spectrometers are employed, with lower mass resolution than the Orbitrap or Q-ToF mass spectrometers used for untargeted analyses. However, these have the advantages of lower cost, higher sensitivity, linearity, and specificity. By employing isotopically labelled standards of the analytes of interest, which are spiked into each study sample, absolute quantification can be achieved. Furthermore, as the analytes being measured are known upfront, and the chromatographic and mass spectrometric methods are optimized at the start, run times can be much shorter than for untargeted analyses. There are guidelines which can be followed for ensuring accuracy and precision of the assay, such as those laid out by the FDA [146]. Software exist for the analysis of targeted data, either vendor provided or freeware such as Skyline [147]. It is likely that as this field of research advances, more targeted assays will be incorporated into the metabolomics workflow.

## 3. Conclusions

Untargeted metabolomics is a powerful approach to understanding changes due to disease, drug treatment, or environmental factors in a multitude of human, animal, and cell models. However, as metabolism is complex, so are the data produced in these studies. It is therefore crucial to be vigilant at every stage of the experiment. If the study has not been designed correctly, it will be hard to elucidate biologically relevant information, as confounding factors may overwhelm any biological changes. To maximize the metabolome coverage, it is necessary to acquire data in several chromatographic and ionization modes, ideally HILIC for polar metabolites and RPLC for complex lipids (using non-polar solvents for elution). MS/MS data—of high quality and volume—can be acquired in DDA mode using iterative injections with PC-driven exclusion and/or in DIA mode with sequential mass windows (e.g., SWATH, SONAR). Furthermore, it is of the utmost importance to pre-process the data correctly, as there will inherently be redundancy in the data. As metabolite identification remains the bottleneck in metabolomics studies, so stringent approaches are needed to ensure that models have been validated and only the strongest candidates are pursued through the identification pipeline. The comprehensiveness of experimentally generated and in silico-derived spectral databases has grown significantly, and their integration into the data processing workflow, together with the improvement of computational approaches (for in silico prediction of MS/MS data), are paving the way towards automated MS/MS data matching to facilitate metabolite annotation. Finally, the advancements in metabolic network analysis tools are enabling more mechanistic insights, beyond the biomarker discovery. Here, metabolite data provide crucial complementary information on “what has indeed happened”, as the phenotype readout at the molecular level, thus representing the “missing piece” of puzzle towards multi-scale omics data integration for more accurate interpretation of biological processes.

## Figures and Tables

**Table 1:** MS/MS data acquisition modes with their advantages and disadvantages.

MS/MS Data Acquisition Mode	Selection of Precursor Ions	Advantage	Pitfall
Selective or targeted MS/MS	Only selected ions specified on an inclusion list will be targeted	Highest quality MS/MS data	A posteriori acquisition, in a separate batch of analyses
Data-Dependent Acquisition (DDA)	Ions are selected for MS/MS acquisition in real-time based on threshold intensity: Top «n» ions are «picked» in each scan Preferred list and exclusion list	High-quality MS/MS data and established link between precursor and product ions	High acquisition rates required. Selection of the most highly abundant ions each time, across multiple scans, resulting in low MS/MS coverage
Data-Independent Acquisition (DIA)	All fragment ions for all precursors are acquired simultaneously: All-ion-fragmentation (Q1 transmits the full mass range, 50–1700 Da of precursor ions in the collision cell: AIF, MS^E^) or with sequential mass windows (Q1 transmits several increments of 20–50 amu across the mass range in the collision cell: SWATH, SONAR, BASIC DIA—see Figure 2)	Improved coverage for low abundant precursor ions	High acquisition rates required. Difficulty of MS/MS data deconvolution to re-establish the link between the precursor and product ions

**Table 2:** Criteria for feature filtering using QC and blank samples in order to reduce data complexity and remove redundancy.

Parameter	Criteria	Outcome	Notes
Coefficient of variation (CV)	Choose threshold of variation, e.g., of metabolite peak area in repeated injections of QC sample	Remove metabolite features, e.g., with CV > 30% in QC samples *	CV cut-off values may be dependent on sample type, chromatography, or instrument parameters
Presence in study samples	Metabolite feature/peak must be present in a certain proportion of the study samples (and/or QCs)	Remove metabolite features present in only a low proportion of study samples	Certain peaks may only be present in one class of samples—adjust threshold accordingly
Presence in blank samples	Metabolite feature/peak must not be present in study samples/at very low levels	Remove metabolite features present in blank samples	Some metabolite features may be present in blank samples due to carryover—ensure multiple blanks have been run to address this
Response to dilution	Metabolite feature/peak must respond to dilution series with r2 > 0.8**	Remove metabolite features with r2 < 0.8 **	Some metabolite features may be saturated at higher concentrations and so do not behave linearly—check raw data

* Some groups recommend a lower cut-off, e.g., 20% [97]; ** this removal should be performed carefully by evaluating the features whose response may not be linear due to their low abundance.

**Table 3:** Major problems and solutions associated with metabolite identification in metabolomic datasets. The references for different tools are cited in the main text.

Bottleneck	Cause	Solutions
*Known metabolite (mis)identification*	Isomers or metabolites with identical mass (and molecular formula) but different structures	Chromatographic resolution (i.e., separation by RT, chiral columns for stereoisomers) Ion mobility MS (IMS and/or cross-collision section—CCS values) MS/MS fragmentation pattern matching against experimentally acquired or in silico generated MS/MS databases (i.e., METLIN, mzCloud, NIST, MassBank, LipidBlast, LipidMaps, GNPS)
	Isobars or compounds of similar molecular weight produce interferences	MS resolution (HRMS using TOF or Orbitrap mass analyzer) Chromatographic resolution (i.e., separation by RT) MS/MS fragmentation pattern matching as specified above Ion mobility MS (IMS and/or cross-collision section—CCS values)
	In-source fragments—due to production of ions (by loss of H_2_O, CO_2_, H_3_PO_4_) that have the same mass and/or structure as the molecular ions of other metabolites	Chromatographic resolution (i.e., separation by RT) MS source with reduced in-source fragmentation
*Unknown metabolite identification*	“*Known unknowns*”—metabolites listed in molecular structure databases but without recorded reference MS/MS spectra in spectral libraries	In silico fragmentation tools and derived databases (e.g., CSI:FingerID coupled to Sirius, MetFrag, iMet, MS2LDA, MS-FINDER, etc.) and similarity matching (of experimentally acquired and in silico generated MS/MS) and network analysis (e.g., GNPS) RT prediction models (limited to specific columns and LC conditions) CCS prediction models and databases (e.g., MetCCS, LipidCCS) Multiple-stage tandem MS (MS^n^)
	“*Unknown unknowns*”—new metabolites not listed in any database	Metabolite isolation and NMR analysis for structural elucidation LC-MS/MS analysis (RT, accurate mass, MS/MS) combined with above indicated tools for “*known unknowns*” Multiple-stage tandem MS (MS^n^)

**Table 4:** List of selected open access web servers for interactive pathway visualization, metabolite mapping, and visualization in the context of pathways and metabolic networks, and metabolite set enrichment and overrepresentation analysis (MSEA, ORA).

**Tool**	**Functionalities**
MeTexplore web server [128]	Metabolite mapping on metabolic pathways and networks Visualizing networks Mining and editing networks based on data and network structure (identify sub-networks connecting identified metabolites) Pathway enrichment analysis Mapping polyomics data Computing fluxes
Pathvisio [129]	Metabolite mapping on the pathways Pathway editing, drawing, and analysis Overrepresentation analysis
iPath—Interactive Pathways Explorer [130]	Metabolite mapping on the pathways Pathway editing and analysis
MetaboAnalyst* web server [91]	Metabolite ID conversion Enrichment analysis (ORA, MSEA) Pathway topology analysis Joint pathway analysis (genes and metabolites) MS peaks to pathways
PathBank [131]	Interactive database for visualizing metabolic pathways in different model organisms Metabolite (as well as gene, protein, drug) search and mapping Detailed description and references are provided for each pathway from energy metabolism, associated with metabolic diseases, drug-action pathways, drug metabolism pathways, signaling pathways
LION/web [132]	Web platform for lipid ontology enrichment analysis Lipid classification by chemical data (LIPIDMAPS), biophysical data, lipid functions and organelle associations
XCMS online* [133]	Activity network analysis i.e., “MS peaks to metabolic network” (integrated *Mummichog* tool) Integrated pathway analysis (using genome and proteome data, in addition to metabolome data)

* Features relevant to pathway and network analysis have been listed here, MetaboAnalyst and XCMS online servers provide plenty of other functionalities related to data processing and analysis.

**Table 5:** List of open access knowledge databases (used in the above listed web servers). Some databases have been extended into pathway browsers for interactive metabolite mapping. Although some databases are gene-centric, all of them are searchable for metabolites and represent a great source of biochemical knowledge for metabolite data interpretation.

Database	Functionalities
KEGG database and pathway browser [139]	Metabolite mapping on metabolic pathways (with annotation of the direction of changes)
Reactome database and pathway browser [140,141]	Visualization of known biological processes and pathways from intermediary metabolism, signaling, transcriptional regulation, apoptosis, disease Metabolite mapping and pathway and network visualization and analysis Pathway enrichment analysis
Cyc databases (EcoCyc, HumanCyc, MetaCyc, BioCyc) [142]	Curated database of experimentally elucidated metabolic pathways from many different model organisms Metabolite, protein, reaction, and pathway search Comparison of specific pathway and metabolic networks of different organisms
Recon database [143,144] Virtual metabolic human	Largest database of human and gut microbiome metabolism Searchable by metabolic reaction, metabolites and genes, by microorganism species, by disease, and by diet Organelle maps
WikiPathways database [145]	Pathway database maintained by scientific community Pathway browsing and editing
